# Prognostic significance of LINC01132 in lung cancer and its regulatory role in tumor progression

**DOI:** 10.1007/s12672-024-00884-7

**Published:** 2024-02-25

**Authors:** Yang Hu, Youying Wei

**Affiliations:** Department of Thoracic Medicine, Hubei Cancer Hospital, No. 116, Zhuodaoquan South Road, Wuhan, 430065 China

**Keywords:** Lung cancer, lncRNA LINC01132, miR-125a-3p, Prognosis

## Abstract

**Background:**

The application of long non-coding RNAs (lncRNAs) in cancer has been the focus of research in recent years. This study aimed to discuss the expression and functional mechanism of lncRNA LINC01132 (LINC01132) in lung cancer and explore its prognostic significance in tumors.

**Methods:**

The expression of LINC01132 in lung cancer patients was verified using GSE98929 screening and real-time quantitative polymerase chain reaction (RT-qPCR) detection. The prognostic potential of LINC01132 was evaluated by performing the chi-square analysis of clinical indicators, Kaplan–Meier analysis, and Cox proportional hazard model. Cell Counting Kit-8 (CCK-8), flow cytometry, and Transwell assay were used to characterize the biological functions of the lung cancer cells. The targeting relationship between LINC01132 and microRNA-125a-3p (miR-125a-3p), miR-125a-3p and SMAD2 was predicted by bioinformatics and verified by luciferase activity assay.

**Results:**

LINC01132 was upregulated in lung cancer tissues and cells, which was an independent risk factor for survival and prognostic outcomes of lung cancer patients. Silencing LINC01132 suppressed the proliferation and migration of lung cancer cells and accelerated cell death. The target of LINC01132 was miR-125a-3p, and miR-125a-3p inhibitor could eliminate the inhibitory effect of LINC01132 knockdown on the cells. Additionally, SMAD2 is a downstream target of miR-125a-3p, and knockdown of SMAD2 reversed the effects of miR-125a-3p inhibitor on cell migration and invasion.

**Conclusion:**

LINC01132 may regulate the progression of lung cancer by targeting the miR-125a-3p /SMAD2 axis and serve as a prognostic biomarker for lung cancer.

## Introduction

Lung cancer is a disease caused by the uncontrolled growth and spread of lung cells. According to the latest data, lung cancer ranks second in incidence in both men and women and is the leading cause of cancer-related death [1]. Over 350 deaths from lung cancer occur each day in the United States, and most of them are related to smoking [2]. China also faced a huge lung cancer burden, wherein the number of new lung cancer cases reached 730,000, as early as 2015, and more than half of them died [3]. Therefore, the prognosis of patients with lung cancer seems poor, especially for patients with non-small cell lung cancer (NSCLC) whose 5-year survival rate is only 15% [4]. Moreover, several lung cancer patients do not present significant symptoms in the early stages of the tumor, which may be overlooked by the patient, resulting in missing the best treatment opportunity. Hence, finding biomarkers to predict survival and prognostic outcomes in lung cancer patients is an arduous task.

The application of molecular targeted therapy in various diseases, such as cancer, has shown the relation between lncRNAs and the molecular mechanism of the disease process. LncRNAs are non-protein-coding transcripts longer than 200 nucleotides [5]. Thousands of lncRNAs associated with tumor development have been identified using transcriptome analysis [6, 7]. LncRNAs may be involved in lung cancer carcinogenesis or drug resistance and have the potential to become biomarkers for lung cancer [8, 9]. For instance, lncRNA MITA1 could serve as a regulator of gefitinib resistance in lung cancer cells [10]. LncRNA HOTAIR has the potential to be a biomarker for NSCLC [11]. LINC01132 is located on chromosome 1q42.3 and regulates pathological mechanisms such as glioblastoma and ovarian cancer [12, 13]. The therapeutic efficacy of LINC01132 in hepatocellular carcinoma was reported by Zhang et al. [14]; however, its role in lung cancer has not been elucidated. Furthermore, lncRNAs interfere with the expression of downstream factors through transcription and protein modification [15]. Therefore, studying the regulatory mechanism of LINC01132 could have of reference significance and clinical value.

Herein, we evaluated the LINC01132 level in lung cancer using bioinformatics screening and sample detection to determine its clinical and prognostic value in lung cancer. Meanwhile, in vitro cell experiments were performed to analyze the regulatory mechanism of abnormal expression of LINC01132 in tumor cells and progression. This study may introduce a new direction for the effective treatment and survival of lung cancer patients.

## Materials and methods

### Lung cancer patients

This study included 117 patients diagnosed with lung cancer from May 2016 to May 2017. Patients with lung cancer were treated with tumor-related treatment before resection and did not have other serious diseases during the same period. The clinical data of the patients were recorded, and the patients were followed up for 5 years for survival. The procedures involved in this study were conducted with the understanding and knowledge of the participants, and the relevant written informed consent was obtained. The Ethics Committee of Hubei Cancer Hospital supervised the study.

Lung cancer tissues and normal tissues adjacent to the tumor were collected after surgery, cooled in liquid nitrogen after cleaning, and frozen in an environment of – 80 °C for further use.

### Culture and transfection of cells

The cells involved in this study, lung cancer cells (H522, H460, SKMES, CAL-12T) and lung fibroblasts (IMR-90), were derived from the ATCC (Rockville, USA). DMEM medium with 10% FBS (Thermo Fisher, USA) were the nutrient sources for the cells. The incubation temperature was set at 37 °C, and the incubator was maintained to contain 5% CO_2_.

Silencing LINC01132 (si-LINC01132), miR-125a-3p mimic/inhibitor, silencing SMAD2 (si-SMAD2) and corresponding negative controls (NC) for transfection assays were designed and provided by Gene Pharma company (Shanghai, China). Cell transfection experiments were all performed using Lipofectamine 2000 (Invitrogen, Carlsbad, CA, USA).

### RNA isolation and PCR assay

Total RNA was isolated from tissues and cells by TRIZOL reagent (Ambion, USA). After verifying the quality of the RNA template, cDNA was obtained by High-Capacity cDNA Reverse Transcription Kit and TaqMan MicroRNA Reverse Transcription Kit (Applied Biosystems, USA). According to the instructions of SYBR^®^GREEN Master Mix (Takara, Japan), the reaction system was configured for RT-qPCR on the ABI 7500 Real-Time PCR system (Applied Biosystems). Furthermore, GAPDH and U6 were normalized for LINC01132 and miR-125a-3p, respectively.

### Cell proliferation and apoptosis assays

Cell Counting Kit-8 (CCK-8; Dojindo, Japan) was used to assess the growth level of the lung cancer cells. Specifically, the cells were seeded in 96-well plates, and the density was adjusted to 5 × 10^3^ cells. Following this, 10 µL of the CCK-8 reagent was transferred to each well at specific time points (0–72 h). After continued incubation at 37 °C for 2 h, the absorbance was read at 450 nm.

Cell death assays were performed using flow cytometry. After washing with PBS, the cells were re-suspended with the buffer in the Annexin V-FITC Apoptosis detection kit (BD Biosciences) and then treated with Annexin V and propidium iodide (PI) in the dark for staining. After 15 min of incubation, the apoptosis rate of the cells was measured by flow cytometry.

### Transwell assays

Cell behavioral assays were performed using the Transwell method. The cells (H522 and H460) and DMEM medium were transferred to the upper chamber of the Transwell (Corning, USA), and DMEM medium containing FBS was added to the lower chamber. After incubation at 37 °C for 48 h, the cells transferred to the bottom were fixed and stained. Finally, cell images were acquired using a microscope (Olympus Corporation, Japan) and counted using NIH ImageJ software. Moreover, the invasive ability was validated using Matrigel (BD Biosciences).

### Online database analysis

The differentially expressed lncRNAs were analyzed using the dataset GSE98929 in the GEO database, and the highly and low-expressed lncRNAs were identified. The abnormally high level of LINC01132 in the lung cancer samples was identified using the GEPIA website. The LncRNASNP2 online tool predicted the downstream target of LINC01132 and confirmed the existence of binding sites between LINC01132 and miR-125a-3p. Meanwhile, TargetScanHuman 7.2 online tool confirmed the targeting relationship between miR-125a-3p and SMAD2.

### Luciferase reporter assay

The wild-type and mutant-type LINC01132 fragments were inserted into the pmirGLO vector (Promega, USA) to generate the LINC01132-WT and LINC01132-MUT plasmids, which were then combined with mimic NC, inhibitor NC, miR-125a-3p mimic, or miR-125a-3p inhibitor and co-transfected into the H522 or H460 cells using Lipofectamine 2000 reagent. SMAD2-WT and SMAD2-MUT plasmids were constructed according to a similar protocol, and co-transfection assays were performed. The luciferase activity of the collected cells was assessed using the Luciferase Reporter Gene Assay Kit (Promega, Germany).

### Survival analysis of the patients

To investigate the prognostic value of LINC01132 in lung cancer, the Kaplan–Meier curve was used to analyze the relationship between the LINC01132 level and the overall survival of patients. Furthermore, Cox multivariate regression analysis was used to evaluate the correlation between patient clinical parameters and lung cancer prognosis.

### Statistical analysis

Statistical analyses were performed using GraphPad Prism 9.0 and SPSS 22.0 software. The chi-square test was used to evaluate the correlation between LINC01132 and clinical indicators of patients. The regulation level of LINC01132 on miR-125a-3p was determined by Pearson analysis. Differences between groups were assessed by one-way ANOVA or Student's *t*-test. The experiment was conducted three times for each group, and *P* < 0.05 was considered statistically significant.

## Results

### LINC01132 levels were elevated in lung cancer

**Fig. 1:**
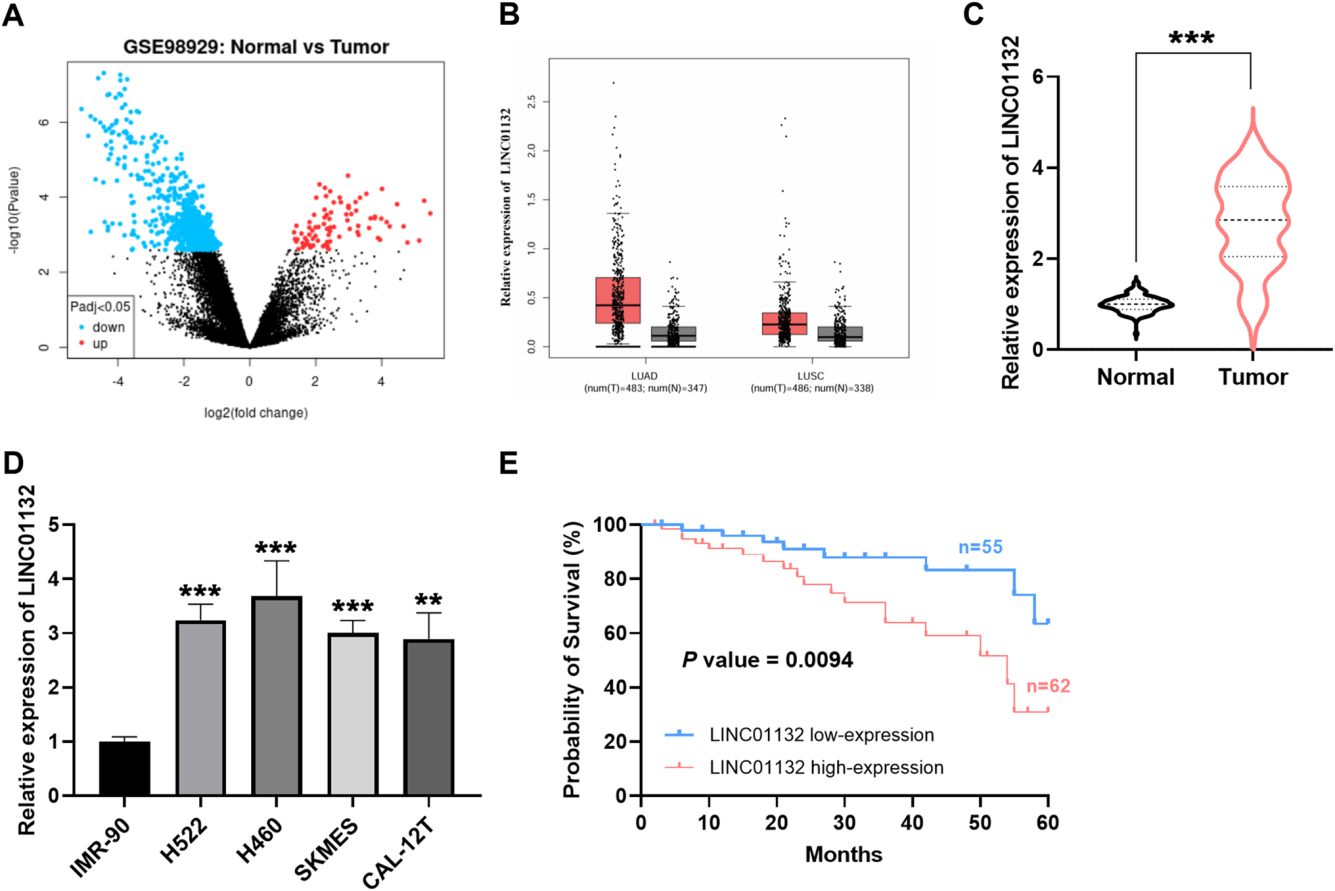
Elevated LINC01132 levels in lung cancer. **A** The GSE98929 dataset is used to screen the differentially expressed lncRNAs. **B** Prediction of LINC01132 expression in lung cancer samples via GEPIA. **C**, **D** LINC01132 is prominently expressed in the lung cancer samples by RT-qPCR assay. **E** The Kaplan–Meier method is used to assess the 5-year survival rate of lung cancer patients (*P* = 0.0094). **P < 0.01, ***P < 0.001

The aberrated lncRNAs were screened in the GSE98929 dataset, and the upregulation of LINC01132 in the lung cancer samples was further confirmed on the GEPIA website (Fig. 1A, B). In our collected tissue samples, LINC01132 was elevated in the lung cancer tissues compared with the adjacent normal tissues (Fig. 1C). Similarly, LINC01132 was also obviously increased in the tumor cells (H522, H460, SKMES, and CAL-12T) in Fig. 1D.

### Clinical function of LINC01132 in lung cancer

The clinical data of 117 lung cancer patients were collated and analyzed as shown in Table 1. Based on the mean expression of LINC01132, the patients were categorized into the high-group and low-group. There were statistically significant associations between LINC01132 with lymph node metastasis (*P* = 0.030) and TNM stage (*P* = 0.007). Furthermore, the clinical prognostic value of LINC01132 in lung cancer was investigated. Figure 1E illustrates the survival outcomes of the patients over 5 years as analyzed by the Kaplan–Meier method (*P* = 0.0094). Additionally, multivariate Cox analysis performed for the clinical characteristics of lung cancer patients revealed that lymph node metastasis (*P* = 0.044), TNM stage (*P* = 0.018), and LINC01132 (*P* = 0.012) were all independent prognostic factors for lung cancer (Table 2). This suggests that elevated LINC01132 has potential as a prognostic biomarker for lung cancer.

**Table 1:** Association of LINC01132 with clinical parameters in lung cancer patients

Parameters	Patients (n = 117)	LINC01132 expression		*P*
		Low (n = 55)	High (n = 62)	
Age				0.802
≤ 60 years	56	27	29	
> 60 years	61	28	33	
Gender				0.506
Male	60	30	30	
Female	57	25	32	
Tumor size				0.054
≤ 5 cm	70	38	32	
> 5 cm	47	17	30	
Differentiation				0.278
Well, moderate	64	33	31	
Poor	53	22	31	
Lymph node metastasis				0.030
Negative	73	40	33	
Positive	44	15	29	
TNM stage				0.007
I, II	61	36	25	
III, IV	56	19	37

**Table 2:** Multivariate Cox analysis of clinical characteristics

Characteristics	HR	95% CI	*P*
Age	1.541	0.577–4.117	0.389
Gender	1.062	0.408–2.763	0.901
Tumor size	0.417	0.164–1.055	0.065
Differentiation	0.506	0.180–1.424	0.197
Lymph node metastasis	0.340	0.119–0.970	0.044
TNM stage	0.163	0.036–0.737	0.018
LINC01132	0.265	0.094–0.745	0.012

### LINC01132 knockdown and in vitro cell assay

**Fig. 2:**
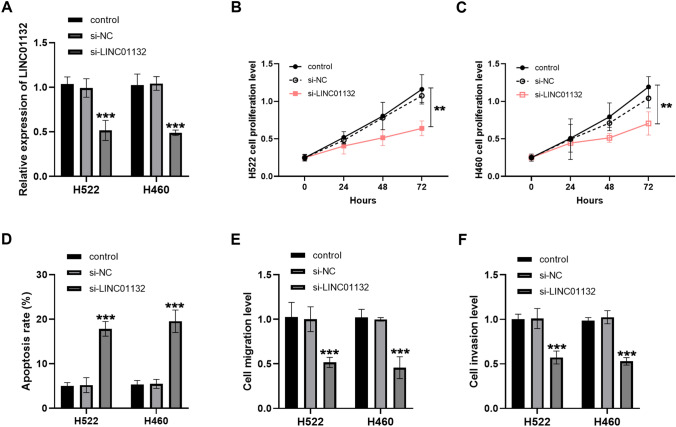
Effect of LINC01132 knockdown on cell viability. **A** Efficiency of transfection of si-LINC01132 in the H522 and H460 cells. **B**–**D** When the LINC01132 level is low, the cell growth ability decreases, while the apoptosis rate increases. **E**, **F** Regulation of LINC01132 silencing on cell proliferation and migration levels. **P < 0.01, ***P < 0.001

We silenced LINC01132 and performed cell transfection, and the transfection results are shown in Fig. 2A. Downregulation of LINC01132 suppressed cell proliferation in Figs. 2B, C. Meanwhile, the apoptosis rate of the cells was enhanced when the LINC01132 expression was low (Fig. 2D). After silencing LINC01132, the number of cell movements reduced significantly (Fig. 2E, F). These results highlighted that LINC01132 knockdown inhibited the viability of the lung cancer cells and aggravated cell death, which led us to speculate that low LINC01132 level might alleviate tumor progression.

### Targeting relationship between LINC01132 and miR-125a-3p

**Fig. 3:**
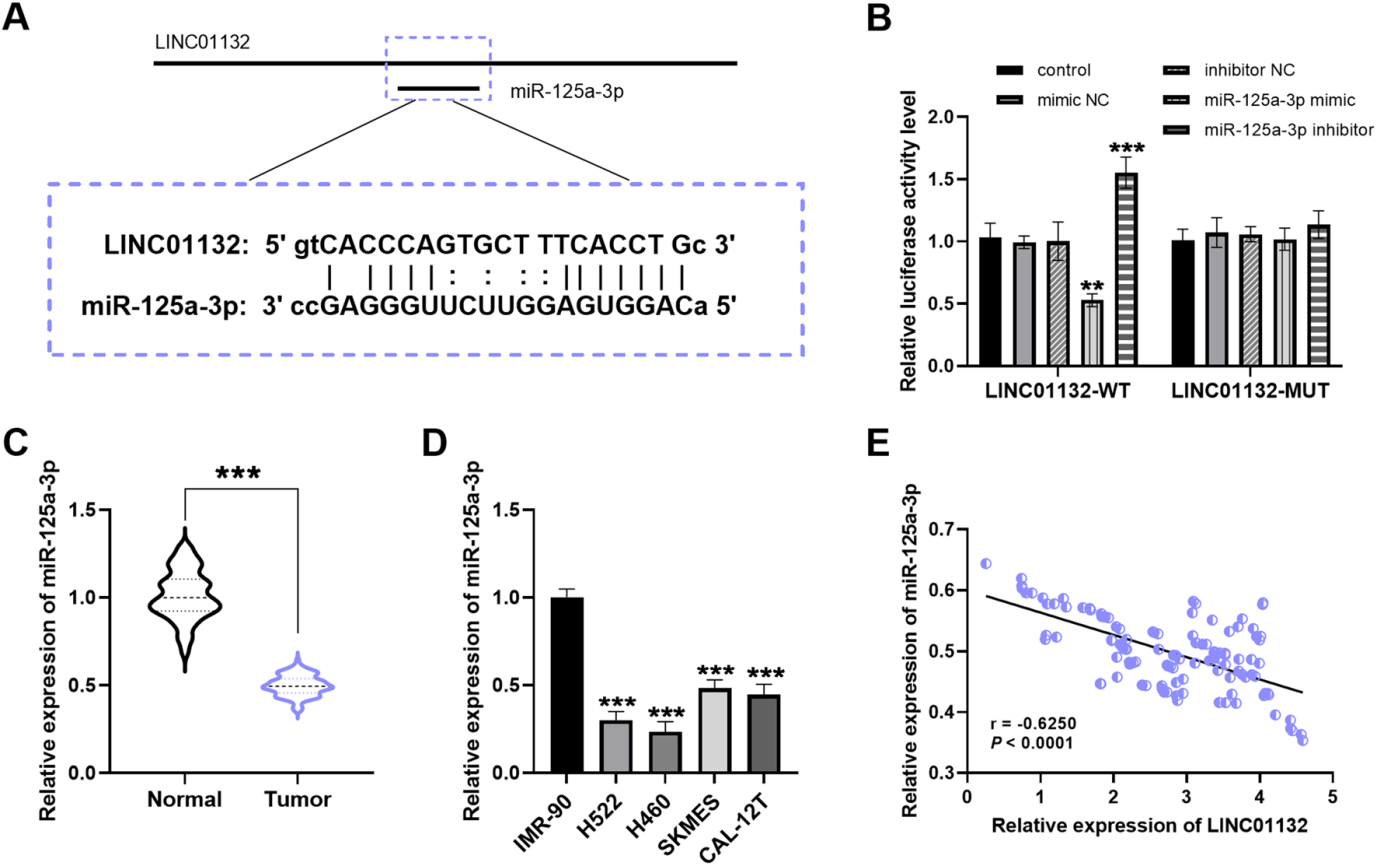
Targeting relationship between LINC01132 and miR-125a-3p. **A** Link sites between LINC01132 and miR-125a-3p. **B** Luciferase activity detection of LINC01132-WT and LINC01132-MUT. **C**, **D** The miR-125a-3p content is down-regulated in the tissues and cells. **E** LINC01132 and miR-125a-3p show an inverse relationship (r = − 0.6250, *P* < 0.0001). ***P < 0.001

To explore the molecular mechanism of LINC01132 in lung cancer more systematically, the binding sites of LINC01132 and miR-125a-3p were predicted using the LncRNASNP2 online tool (Fig. 3A). In addition, miR-125a-3p mimic/inhibitor was verified to alter the luciferase activity of LINC01132-WT, but it did not affect LINC01132-MUT (Fig. 3B). The downregulation of miR-125a-3p in tumor tissues (Fig. 3C) and cells (Fig. 3D) was elucidated by RT-qPCR. The miR-125a-3p level correlated negatively with LINC01132 in the lung cancer tissues (r = − 0.6250, *P* < 0.0001; Fig. 3E). Thus, LINC01132 acted as a sponge for downstream miR-125a-3p, which inversely regulated miR-125a-3p.

### Regulation of cells by silencing LINC01132 and miR-125a-3p inhibitor co-transfection

**Fig. 4:**
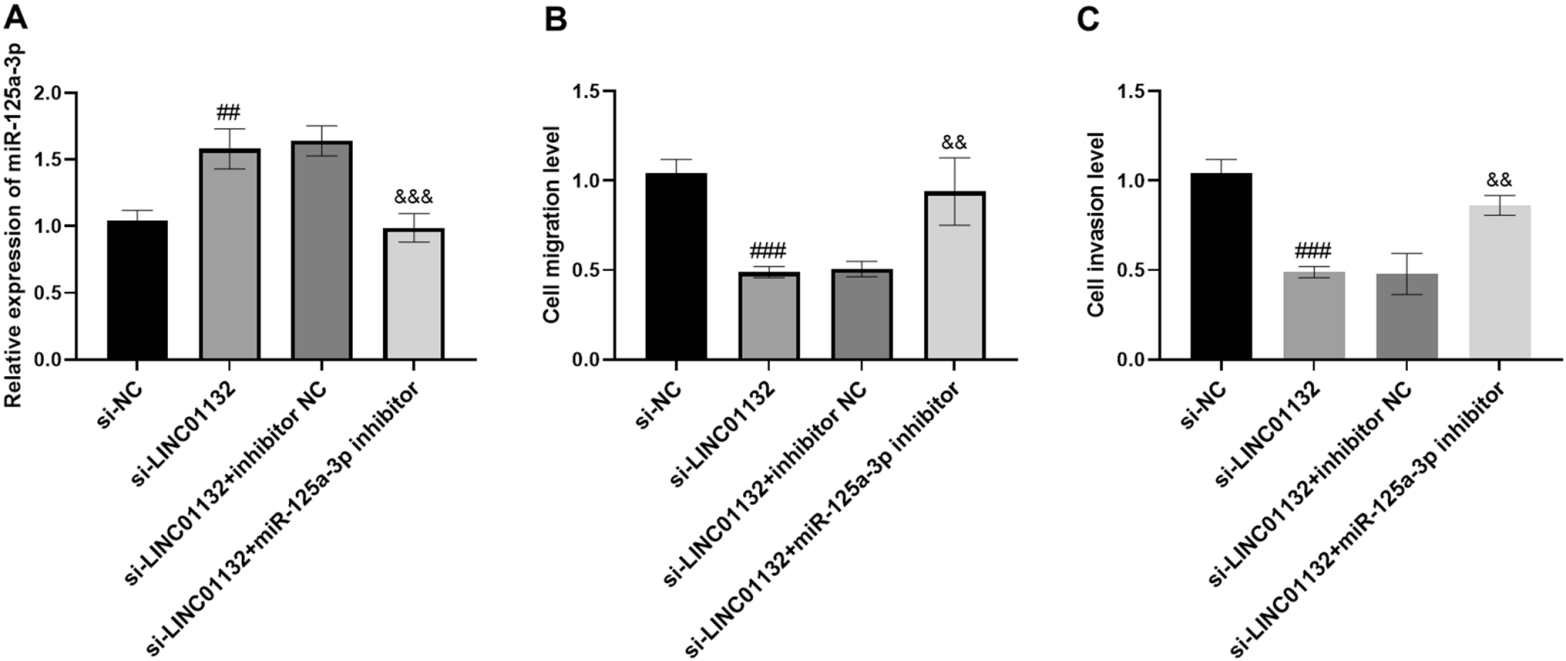
Regulation of cell biological behavior by miR-125a-3p inhibitor. **A** The content of miR-125a-3p in the cells after transfection with si-LINC01132 and miR-125a-3p inhibitor. **B**, **C** miR-125a-3p inhibitor counteracts the inhibitory effect of si-LINC01132 on cell migration and invasion. ^##^*P* < 0.01, ^###^*P* < 0.001, with si-NC; ^&&^*P* < 0.01, ^&&&^*P* < 0.001, with si-LINC01132

We verified the targeted regulation relationship between LINC01132 and miR-125a-3p by cell behavioral reversion assay. Figure 4A shows that the miR-125a-3p level increased after transfection with si-LINC01132, while miR-125a-3p decreased after co-transfection with si-LINC01132 and miR-125a-3p inhibitor. Transwell assay also confirmed that the involvement of miR-125a-3p inhibitor could restore the amount of cell migration (Fig. 4B) and invasion (Fig. 4C). Thus, LINC01132 affects the progression of lung cancer by mediating the miR-125a-3p expression.

### Regulation of cell biological activity by knockdown of SMAD2

**Fig. 5:**
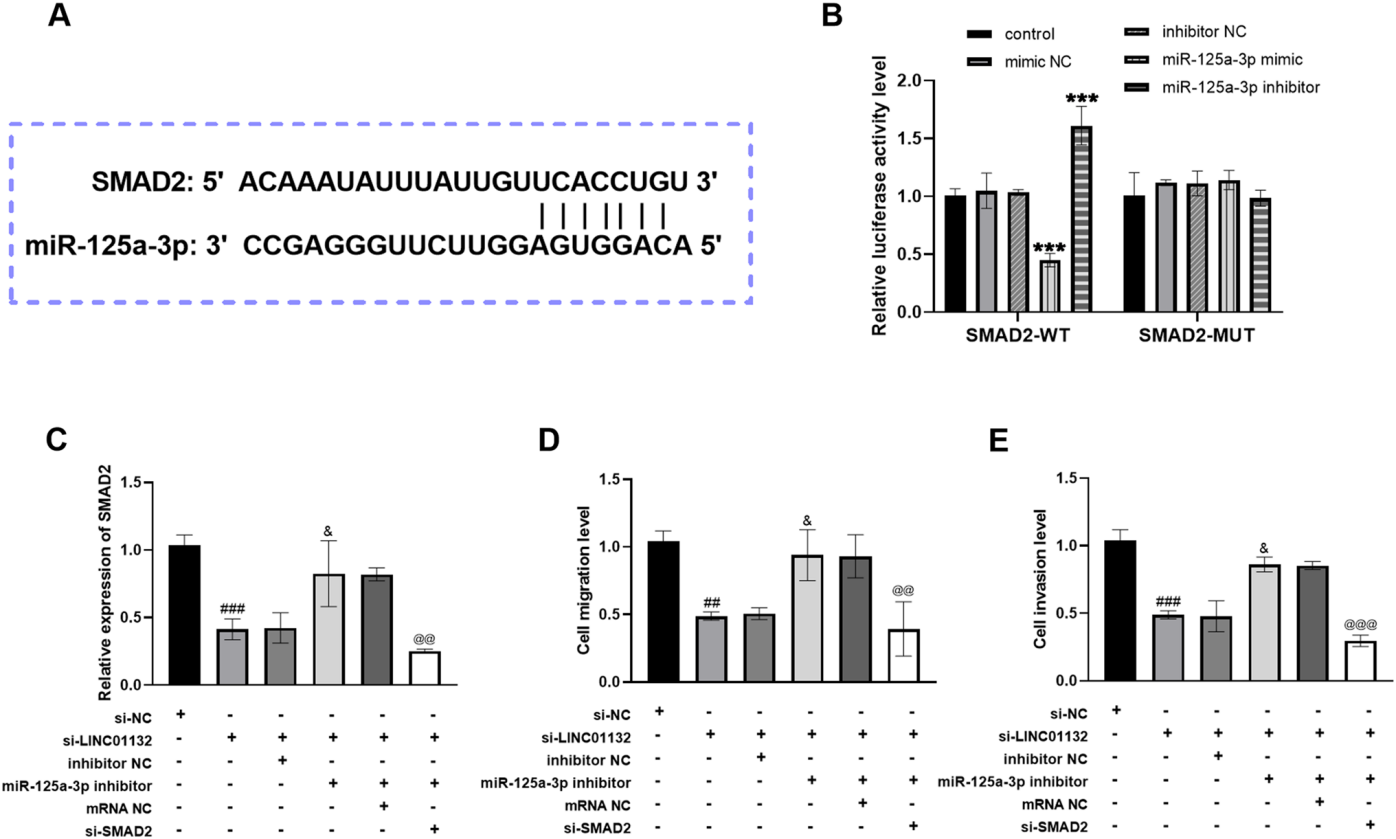
Effect of LINC01132 on miR-125a-3p/SMAD2 axis. **A** Binding sites between miR-125a-3p and SMAD2. **B** Luciferase activity detection of SMAD2-WT and SMAD2-MUT. ***P < 0.001, with control. **C** Changes in SMAD2 expression after transfection with si-LINC01132, miR-125a-3p inhibitor and si-SMAD2. **C**–**E** Regulation of SMAD2 knockdown on the biological function of lung cancer cells. ^##^*P* < 0.01, ^###^*P* < 0.001, with si-NC; ^&^*P* < 0.05, with si-LINC01132; ^@@^*P* < 0.01, ^@@@^*P* < 0.001, with si-LINC01132 + miR-125a-3p inhibitor

Furthermore, SMAD2 was found to have binding sites with miR-125a-3p by predicting the downstream targets of miR-125a-3p (Fig. 5A). Luciferase activity assay demonstrated that miR-125a-3p directly targets SMAD2 (Fig. 5B). Figure 5C depicts the decreased SMAD2 level in lung cancer cells after transfection with si-LINC01132 compared with the si-NC group, while the involvement of miR-125a-3p inhibitor reversed the inhibition of SMAD2 level by silencing LINC01132. Interestingly, the promotion effect of miR-125a-3p inhibitor on SMAD2 levels was counteracted after the introduction of si-SMAD2. Transfection of si-SMAD2 restored the enhancing effect of miR-125a-3p inhibitor on cell migration and invasion ability (Fig. 5D, E).

## Discussion

The survival outcome of patients with lung cancer after surgery is concerning, and the underlying mechanisms are unclear. To understand the molecular mechanism of lung cancer systematically, we conducted clinical and cellular-related studies using LINC01132 as an example to find the perfect and effective prognostic measures.

In our study, LINC01132 was screened using GSE98929 and GEPIA to be upregulated in lung cancer, and the elevated level of LINC01132 in the tissues and cells was confirmed by RT-qPCR assay. In the discussion of lncRNAs, evidence suggested that lncRNAs are highly expressed in lung cancer and are regarded as valuable therapeutic factors [16–18]. LINC01132 was also upregulated as a cancer-promoting factor in epithelial ovarian cancer and predicted poor prognosis [19]. Similarly, we observed that elevated LINC01132 was associated with poor survival outcomes in lung cancer patients and was an independent prognostic factor for lung cancer. This suggests that lung cancer patients with high expression of LINC01132 have poor prognosis and that LINC01132 may play a regulatory role in the progression of lung cancer. To evaluate the function of aberrant LINC01132 expression further, we selected appropriate lung cancer cells for the transfection assay. The results of in vitro cell experiments showed that the growth and motility of the lung cancer cells were significantly suppressed when LINC01132 was downregulated, while the level of apoptosis increased. Previous studies have suggested that high expression of LINC01132 has positive implications on the growth and metastasis of hepatocellular cancer cells, and LINC01132 knockdown mediates the therapeutic effect by affecting cell infiltration [14]. Therefore, we believed that silencing LINC01132 would slow down tumor progression by controlling the cellular behavioral state.

The underlying mechanism of LINC01132 in lung cancer was investigated using the cell function assays. Bioinformatics prediction showed that LINC01132 could act as a sponge for specifically binding downstream miR-125a-3p, which was subsequently confirmed by the luciferase activity assay. miR-125a-3p decreased in the tissues and cells in lung cancer, which correlated negatively with LINC01132 in this study. Additionally, miR-125a-3p is involved in various diseases and is downregulated in patients with psoriasis, papillary thyroid cancer, and rheumatoid arthritis, which corroborated our study results [20–22]. Crucially, miR-125a-3p has long been shown to be reduced in lung cancer [23, 24]. Wan et al. focused on circ_0002483 accelerating lung adenocarcinoma progression by targeting miR-125a-3p, while miR-125a-3p mimic counteracted the cancer-promoting effect of circ_0002483 [25]. Tong et al. also observed that TP73-AS1/miR-125a-3p/ACTN4 regulatory network could mediate the occurrence and development of NSCLC, and miR-125a-3p inhibitor reversed the inhibitory function of silencing TP73-AS1 on tumor growth [26]. In this study, we illustrated that the migration and invasion levels of cells were restored after transfection with miR-125a-3p inhibitor. Therefore, the aforementioned studies indicate that silencing LINC01132 directly targets miR-125a-3p to regulate the progression of lung cancer, while inhibition of miR-125a-3p eliminates the impact of LINC01132 knockdown on lung cancer metastasis.

Moreover, miR-125a-3p participates in the development of cancer by mediating downstream factors, such as FOXM1and IL-21R [27, 28]. In the present study, SMAD2 was found to be a target of miR-125a-3p. The introduction of silencing SMAD2 reversed the effect of miR-125a-3p inhibitor on the biological activity of lung cancer cells, suggesting that LINC01132 may participate in the progression of lung cancer by mediating the miR-125a-3p/SMAD2 axis. It cannot be ignored that there are still some limiting factors reflected in this study. On the one hand, the included sample size was limited, and there was a lack of relevant animal experiments. On the other hand, the research methods are relatively single, which limits the breadth and depth of the research. We will improve the reliability and depth of the conclusion with more comprehensive consideration and design in future research.

In summary, we evaluated the expression and functional mechanism of LINC01132 in lung cancer, and it was confirmed that LINC01132 is a prominently expressed lncRNA in lung cancer and has extensible potential value as a prognostic biomarker. Moreover, from the perspective of molecular mechanism, LINC01132 mediates tumorigenesis by targeting the miR-125a-3p/SMAD2 axis.

## Data Availability

The datasets used and/or analysed during the current study are available from the corresponding author on reasonable request.
